# Current News

**Published:** 1897-05

**Authors:** 


### Current News.

### MEETING OF THE AMERICAN MEDICAL ASSOCIATION.

The semi-centennial meeting of the American Medical Association will be held in Philadelphia, Pa., June 1–4, 1897.

Much interest centres around it, since fifty years ago this Association held its first meeting in the city of Philadelphia.

Unusual preparations are being made to have it the most important meeting in the history of the Association.

The officers of the Dental and Oral Section invite the dental profession to attend this meeting and take part in the discussion of the papers. Those wishing to become members can do so by obtaining credentials from their State or local dental society and presenting them with the sum of $5.00 to the secretary.

The programme is here presented.

### PROGRAMME OF THE DENTAL AND ORAL SECTION OF THE AMERICAN MEDICAL ASSOCIATION.

Chairman's Address . . . . . . . . . . . . . . . . Dr. R. R. Andrews, Cambridge, Mass.

A Plea for Conservative Oral Surgery, with Practical Illustrations . . . . . . . . . . . . . . . . Dr. G. Lenox Curtis, New York City, N. Y.

Sterilized Roots of Beasts' Teeth as Supports for Porcelain Crowns . . . . . . . . . . . . . . . . Dr. W. E. Walker, Pass Christian, Miss.

Tumors of the Maxilla . . . . . . . . . . . . . . Dr. Wm. Knight, Cincinnati, Ohio.

The Influence of Diseased Teeth upon the Adjacent Structures . . . . . . . . . . . . . . . . . . . Dr. J. Taft, Cincinnati, Ohio.

Etiology and Treatment of Inflammation of the Anterior Pillars of the Fauces . . . . . . . . . Dr. G. T. Carpenter, Chicago, Ill.

Pathologic Conditions of the Throat and Contiguous Structures during Early Childhood, Prime Factors in the Causation of Irregularities of the Maxilla and Teeth . . . . . . . . . . . . . . . Dr. Wm. A. Mills, Baltimore, Md.

Dental Faculties in Medical Schools . . . . . . . Dr. Richard Grady, Baltimore, Md.

The Need of Dental Instruction in Medical Schools Dr. Edward Branigan, Boston, Mass.

A Series of Clinical Cases . . . . . . . . . . . . Dr. Vida A. Latham, Chicago, Ill.

Hyperkinesis of the Muscles of Mastication a Symptom and an Etiological Factor in Nervous Affections, particularly Neuralgia of the Trigeminus, and Diseases of the Jaws . . . . . . . . . . . . Dr. G. V. I. Brown, Duluth, Minn.

The Relation of Dentistry to General Medicine . . Dr. Geo. F. Eames, Boston, Mass.

Cataphoresis in the Use of Electricity alone for Obtunding Sensitive Dentine. . . . . . . . . . . . Dr. W. G. A. Bonwill, Philadelphia, Pa.

Fraternity . . . . . . . . . . . . . . . . . . . . . Dr. A. C. McCurdy, Towson, Md.

Pyorrhœa Alveolaris in Mercurial and Lead Poisoning and Scurvy. Paper No. 4 . . . . . . . . . . Dr. E. S. Talbot, Chicago, Ill.

R. R. ANDREWS, Cambridge, Mass.,
*Chairman.*

EUGENE S. TALBOT, Chicago, Ill.,
*Secretary.*

---

### RESOLUTIONS OF AMERICAN ACADEMY OF DENTAL SCIENCE.

THE Academy, viewing with dismay the character of the advertisements appearing in some of the self-styled dental journals, whereby secret preparations, often of a highly dangerous character, are paraded in such company and guise as to deceive those not accustomed to scrutinize closely all medicines thus offered, and more particularly of advertisements soliciting dentists to advertise, announcing that "professional dignity and good advertising will work well together," giving the name and address of the professional "writer of dentists' advertisements," and the unscrupulous acceptance of the above-mentioned journals of advertisements, the character of which is detrimental in the highest degree to the advancement of our profession, the best element of which is striving with self-sacrificing and untiring labor to make it worthy the name and title of a liberal and learned profession; therefore,

*Resolved*, That the Fellows of the American Academy of Dental Science strongly condemn such advertising, believing that it is degrading and injurious to the good name of the honorable calling they represent; and they further declare that the editors of such journals, allowing the common tricks of trade to dominate that which should be governed by professional dignity, are unworthy to be acknowledged as teachers and respected confrères among dentists.

*Resolved*, That this resolution be forwarded to the editors of the leading dental journals, as expressing the sentiment of the Academy.

---

### BOARD OF REGISTRATION IN DENTISTRY.

A MEETING of the Massachusetts Board of Registration in Dentistry for the examination of candidates will be held in Boston, Monday, June 14, 1897, at ten A.M., at Dr. Dowsley's office, 175 Tremont Street. Hereafter the board will hold three meetings a year,—in June, November, and March.

E. V. McLEOD,
*Secretary.*

NEW BEDFORD, MASS.

---

### MICHIGAN DENTAL ASSOCIATION.

THE annual meeting of the Michigan Dental Association will be held this year at Battle Creek, on June 8, 9, and 10.

HENRY C. RAYMOND, D.D.S.,
*Secretary.*

### RECENT PATENTS.

A LIST of recent dental patents reported for the INTERNATIONAL DENTAL JOURNAL:

This is the list of dental patents issued March 16, 1897.

No. 577,507.—Dental plugger. Alfred Cane, Golden Gate, Cal. Filed June 11, 1895.

No. 577,638.—Furnace for dental use. John T. Barker, Wallingsford, Conn. Filed June 1, 1896.

No. 577,674.—Inhaler. Theodore B. Wilcox, Newark, N. J. Filed June 1, 1896. Assigned to Sharp and Dohme, New York, N. Y.

No. 577,681.—Inhaler. Harley M. Dunlap, Battle Creek, Mich. Filed January 4, 1896.

No. 577,682.—Syringe. Frederick Eissner, New York, N. Y. Filed January 30, 1896.

No. 578,729.—Dental clamp. J. Austin Dunn, Chicago, Ill. Filed January 15, 1896.

No. 579,094.—Dental chair. Mont C. Merker, Philadelphia, Pa. Filed October 28, 1896.

Design.—No. 26,678.—Dental tool for carrying amalgam. James W. Ivory, Philadelphia, Pa. Filed June 12, 1896. Term of patent fourteen years.

Trade-Marks.—No. 29,839.—Tooth-paste. Walter Benney, New York, N. Y. Filed December 15, 1896. Essential feature: The word "Regal" associated with a medallion and portrait. Used since July 15, 1896.

No. 29,840.—Dentifrice. Horace C. G. Luyties and F. A. Luyties, St. Louis, Mo. Filed October 31, 1896. Essential feature: The word "Sanitol." Used since August 15, 1896.

No. 29,841.—Dentifrices and preparations for teeth and gums. Purity Dental Specialty Company, Sullivan, Ind. Filed February 5, 1897. Essential feature: The representation of a calla lily in connection with the word "Purity." Used since August 16, 1896.

